# Assessment of Water Mobility in Surf Clam and Soy Protein System during Gelation Using LF-NMR Technique

**DOI:** 10.3390/foods9020213

**Published:** 2020-02-19

**Authors:** Siqi Wang, Rong Lin, Shasha Cheng, Zhixiang Wang, Mingqian Tan

**Affiliations:** 1 School of Food Science and Technology, Dalian Polytechnic University, Dalian 116034, China; wangsiqi93dpu@163.com (S.W.); lr131789@163.com (R.L.); chengshasha880321@126.com (S.C.); 2 National Engineering Research Center of Seafood, Dalian 116034, China; 3 Collaborative Innovation Center of Seafood Deep Processing, Dalian 116034, China; 4 Engineering Research Center of Seafood of Ministry of Education of China, Dalian 116034, China; 5 College of Engineering/National R&D Center for Chinese Herbal Medicine Processing, China Pharmaceutical University, Nanjing 211198, China; chinawzx@sohu.com

**Keywords:** myofibrillar protein, soy protein, gelation, LF-NMR

## Abstract

Water mobility and distribution of a dual-protein system of surf clam myofibrillar protein (MP) and soy protein (SP) was investigated by the nondestructive low field nuclear magnetic resonance (LF-NMR) technique. Four proton populations were found in the contour plots of T_2_ relaxation times for the SP-MP system. The first component, (T_21_), was assigned to the highly integrated water located in protein macromolecules with a relaxation time of approximately 1.15 ms. The second signal, T_22_, with a relaxation time of 2.20 to 38.00 ms was regarded as the inter-myofibrillar water trapped in organized protein structures. The third component, T_23_, with a relaxation time of around 100 ms was ascribed to the extra-myofibrillar water. With an increase in temperature, T_24_ appeared which was assigned to the free water within the extra-myofibrillar space. The gelation behavior occurred at 70, 62, and 52 °C as the proportion of SP/MP was 4:6, 2:8, and 0:10, respectively. The principal component analysis (PCA) and heatmap of LF-NMR data analysis showed potential for distinguishing the different dual-protein systems formed at various temperatures. The analysis of storage modulus G′, loss modulus G″, and tanδ confirmed the change trend of the LF-NMR results. The measurements of cooking loss, water holding capability, and gel strength further revealed that the SP and MP were likely to form a gel network with an increase of additional clam protein. The hydrophobicity analysis showed, for the systems with the SP/MP proportions of 4:6, 2:8, and 0:10, more hydrophobic groups were exposed when the temperature was over 50 °C. Scanning electron microscopy showed that the number of the micropores increased with an addition of MP in the dual-protein system of SP/MP. All the results demonstrated that LF-NMR has great potential for characterizing the gelation process of a dual-protein system.

## 1. Introduction

As one of the important edible shellfishes, Chinese surf clam (*Mactra chinensis*) is a famous economic bivalve species with high nutritive value, which is distributed along the coast of Bohai and Huanghai, China [1]. The surf clams are known for their delicate and sweet taste with a high content of myofibrillar protein (MP), a type of protein with important biological functions in the body. It is well-known that the MP plays a pivotal role in determining protein properties of food products, such as water holding capacity (WHC), production yield, and texture of sausage products [2,3,4]. Heating is usually involved in preparing MP products, and the thermal-induced gelation of MP leads to gel formation with high strength. MP molecular rearrangement results in the formation of a network structure after heating [5]. Moisture mobility is an important factor that affects the formation of hydrothermal gels of MP, thus, changing the properties of meat products. It has been reported that the MP is separated and partially unfolded in the early period of a heat-induced gelation process before assembling into gel [6]. Therefore, monitoring the water mobility of MP during gelation is highly desirable. 

Dual-protein food is a new conception that provides a well-balanced diet for solving problems of nutritional deficiency and nutrition imbalance. Plant protein, such as soy protein (SP), has been widely used as a gelling additive in meat products increasing the production yield and texture by enhancing the water-combination property [7]. It is possible to make a dual-protein food item by combining the advantages of surf clam MP and SP. Water mobility and protein play important roles in stabilizing the moisture in a three-dimensional network structure of thermal-induced gel and improve the quality of meat products [8]. During gelation, the denaturalization of proteins influences the mobility and distribution, and thus changes the nature of the gel. Rawdkuen et al. [9] found that a more compact protein network of kamaboko gel could be obtained by acid shift. Puolanne et al. [10] reported that the swelling of MP enhances the interaction between the biomacromolecules and affects the bulk water retaining capacity of gelation systems. Yang et al. [11] found that the proton mobility state is highly related to both water and fat molecules in pork batter during thermal-induced gelation. The analysis of water states is helpful in monitoring the gel change during the gelation process of food products [12,13]. Water mobility is monitored by measuring the relaxation time with the low field nuclear magnetic resonance (LF-NMR) technique. LF-NMR has been proven to be a powerful method for monitoring proton migration and identifying the distribution of water components during food processing, which can characterize the proton (mainly from water in food items) relaxation of spin-lattice (T_1_) and spin-spin relaxation time (T_2_) [8]. The relaxation times are closely related to protein denaturation, water holding ability, surface area, and structure changes [12,13]. The water mobility in a food system can be nondestructively measured via monitoring the relaxation time changes in an external permanent magnetic field by LF-NMR [13]. LF-NMR provides dynamic information of the water states and distribution during food processing, which is very helpful in understanding the interaction mechanism of complex food components. However, the assessment of moisture mobility and redistribution for surf clam MP and SP during gelation is rarely reported.

The purpose of this study is to investigate the interaction of surf clam MP and SP in making a dual-protein food system through monitoring moisture state change, and to obtain the information of degeneration reaction of proteins during the gelation process. The denatured transition between various compositions of mixed protein was analyzed via in situ variable-temperature LF-NMR. Meanwhile, cooking loss, gel strength, and WHC of the gels were also investigated to examine the influence of MP in the mixed gel system. These results revealed that protein interaction with moisture molecules affects the gel properties and microstructure in the protein system. 

## 2. Materials and Methods 

### 2.1. Materials

Soy protein (SP) powder with a purity of 95% was bought from Beijing Aoboxing Biotechnology Co. Ltd. (Beijing, China). Surf clams were obtained from a local market (Liujiaqiao street, Dalian, China). The clams were soaked in NaCl aqueous solution for 30 min and washed with deionized water before collecting the clam meat. Chemical reagents (Na_2_HPO_4_, NaH_2_PO_4_, and HCl) were of analytical grade and purchased from Damao (Tianjin, China).

### 2.2. Preparation of Clam MP and SP Solution

Extraction of clam MP was performed according to the protein extraction method of Cao [14] with minor modification. The clam muscle (100 g) was ground into a pulp using a mincing machine and homogenized with phosphate buffer solution (PBS, 0.05 mol/L, pH 7.0) 5 times. The mixed sample was centrifuged at 10,000 r/min for 10 min. Then, the insoluble substance was collected, added 5 times to PBS buffer (0.05 mol/L, pH 7.0), and centrifuged at 10,000 r/min for 10 min to remove water-soluble protein. The supernatant was stirred for 24 h with PBS buffer (0.05 mol/L, pH 7.0) and NaCl solution (0.6 mol/L) and centrifuged at 10,000 r/min for 20 min to obtain the MP. The final content of MP in the solution was 3.6% *w*/*w*. In addition, the SP powder (3 g) was added into 100 mL of water and stirred overnight. The final concentration of SP solution was 7.8% *w*/*w*. All the operations were conducted below 4 °C condition. 

### 2.3. Preparation of Protein Gels 

**Scheme 1:**
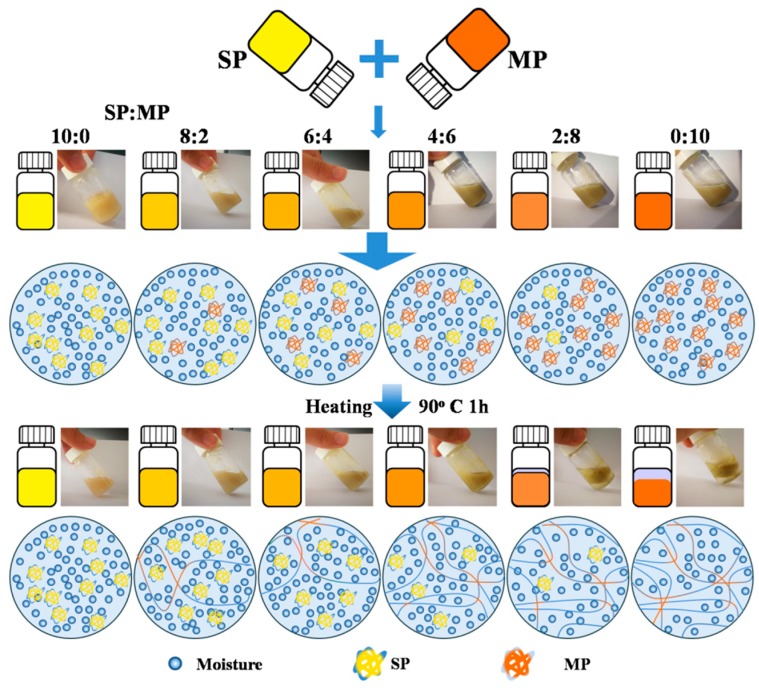
Schematic illustration of preparing the double-protein gel with soy protein (SP) and myofibrillar protein (MP) before and after heating at 90 °C for 1 h.

Two grams of mixed protein (MP and SP) were added into 5 mL PBS buffer (0.05 mol/L, pH 7.0) and stirred vigorously to obtain a homogenous dispersion system. Dual-protein gel was prepared by mixing the MP and SP dispersion varying the percentages of SP mixed with diluted MP (500 mL) as SP:MP = 10:0, 8:2, 6:4, 4:6, 2:8, and 0:10 (*wt*/*wt*). The samples were equilibrated at room temperature before measurements. Scheme 1 shows the experimental design, photographs of protein gel, and the schematic diagram of the protein structure changes before and after 90 °C heating for 1 h.

### 2.4. Relaxation Properties of Protein Gelation

A variable-temperature MesoMR23-060V-1 LF-NMR analyzer from Suzhou Niumag Analytical Instrument Co., (Suzhou, China) was used to measure the relaxation properties with proton resonance at a frequency of 21 MHz. The gel sample was put in a 2 mL transparent plastic cube and tested with a 40 mm diameter tube at 32 °C of the magnets. Carr-Purcell-Meiboom-Gill (CPMG) sequence was used to measure spin-spin relaxation time (T_2_) [15,16], and the delay data were acquired from 5000 echoes with 4 scan repetitions. The samples were gradually heated by hot air in situ with an electrical heater. The protein solution was heated from 38 to 90 °C with 2 °C increments.

The LF-NMR relaxation curve was converted into a multi-index fitting curve using MultiExpInv Analysis Software from Suzhou Niumag Analytical Instrument Co., Suzhou, China. All measurements were carried out in triplicate. A multi-index fitting analysis was carried out on the LF-NMR relaxation data. The peak time constant of the relaxation process was determined by peak location, and the peak area was cumulated from integration of the corresponding peak signal values of the unit time [17]. The contour plots were developed by plotting the relaxation time for the X-axis, and the corresponding T_2_ relaxation signal at various temperatures for the Y-axis using the software Origin 8.5 (OriginLab Corporation, MA, USA). The LF-NMR relaxation curves *M(t)* were fitted by the following equation:

(1) $$M\left(t\right)=\sum_{n=1}^{N}M_{0,n}\exp\left(\frac{-t}{T_{2,n}}\right)+e\left(t\right),$$

where *M(t)* represents the residual magnetization at time t, *M_0,n_* is defined as the magnitude parameter of the n^th^ exponential, *T*_2,n_ represents the transverse relaxation time constant, and *e(t)* is the residual error [18]. 

### 2.5. Rheological Analysis

The dual-protein samples were tested using a Discovery HR-1 rheometer of Advanced Rheometric Expansion System from TA Instrument (New Castle, DE, USA) to obtain the storage modulus during the heating process. The protein samples were placed on 30 mm diameter plates with 1 mm gap. During the temperature scanning, the temperature rose from 38 °C to 90 °C at a rate of 1 °C /min with a strain of 2%. Pre-experimental work showed that a strain of 2% was within the linear viscoelastic region. G’ (storage modulus) and G” (loss modulus) were obtained to showed viscoelastic properties. 

### 2.6. Cooking Loss and Gel Strength Analysis

The protein solution was placed in 2 mL plastic vials and heated in a water bath at 90 °C for 1 h. The squeezed-out fluid was removed from the gel to calculate the cooking loss. The residual gelatinous samples were immediately chilled at 4 °C in a refrigerator. The samples were equilibrated at room temperature for 1 h before further analysis. Gel strength was tested using a Texture Analyzer (TA.XT Plus, Stable MicroSystems, London, UK) with a loading cell of 5 kg. The measurement was performed with a P/0.5 cylindrical stainless-steel probe to a penetration depth of 5 mm at 1 mm/s speed. The measurements were carried out three times for each treatment.

### 2.7. Hydrophobicity of the MP

To investigate the hydrophobicity of the MP and SP mixed system, 8 mmol/L 8-anilino-1-naphthalenesulfonic acid (ANS) was used as a fluorescent indicator by incorporating it into 1 mg/mL concentration protein with PBS buffer. Fluorescence intensity was recorded using an F-2700 fluorescence spectrophotometer (PerkinElmer, Waltham, MA, USA) at the excitation wavelength of 390 nm and the emission wavelength of 470 nm. The protein S0 (surface hydrophobicity) was expressed by the slope of fluorescence intensity to protein concentration.

### 2.8. WHC

The test of WHC was performed as reported by Salvador et al. [19] with minor modification. The gel sample (0.5 g) was placed in a 2 mL centrifuge tube and centrifuged at 6000 r/min at 20 °C. The excess water was removed from the gel with filter paper. WHC was calculated as gel weight after centrifugation, divided by the original mass of the gel and multiplied by 100%.

### 2.9. Microstructure of the Gel 

The gel samples were cut into small cubes with dimensions of 2 × 2 × 1 mm and incubated in 2.5% *v*/*v* glutaraldehyde solution for 2 h. After being washed with distilled water to remove the residual glutaraldehyde. the cubes were soaked in a series of ethanol concentrations (50, 70, 80, 90, and 100%) for 10 min each. The cubes were, then, rinsed with 100% ethanol 2 times and, then, dried at room temperature. The dried gel samples were attached on the platform with graphene (Agar Scientific Ltd. Unit, London England), and coated with 15 nm gold. A high-resolution image of the gel was observed using a scanning electron microscope (SEM, SU8010, Hitachi, Tokyo, Japan) with a working distance of 10,000 μm, and an accelerating voltage of 15 kV. The SEM images were exported with system-provided image processing software of Windows.

### 2.10. Statistical Analysis

One-way analysis of variance (ANOVA) was performed to estimate the significant difference between datasets using SPSS 16.0 software from SPSS Inc. (Chicago, IL, USA). All the images were plotted with Origin 8.5 software (OriginLab Corporation, MA, USA). Principal component analysis (PCA) was processed with Unscrambler 9.7 (CAMO, Norway) to distinguish the difference in the gel system.

## 3. Results

### 3.1. Moisture Mobility Analysis with LF-NMR

**Figure 1:**
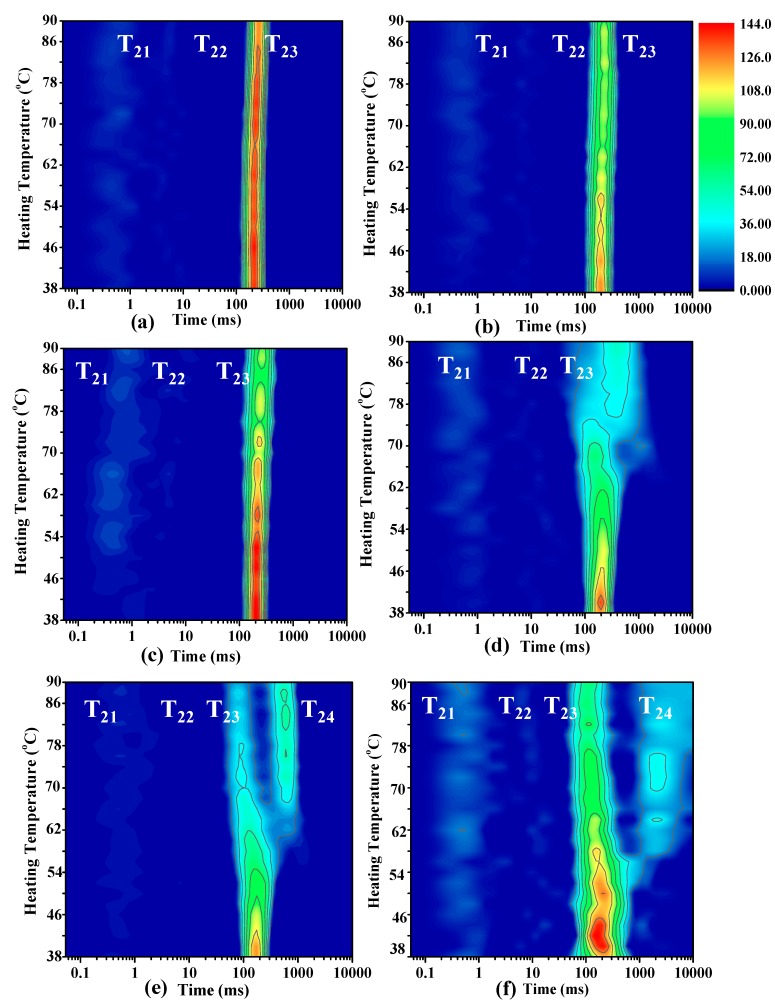
Plots of T_2_ relaxation times for the SP and MP system during heating from 38 to 90 °C with SP: MP ratio at 10:0 (**a**); 8:2 (**b**); 6:4 (**c**); 4:6 (**d**); 2:8 (**e**); and 0:10 (**f**). The initial protein content of SP and MP solution was 7.8% and 3.6%, respectively.

In the thermal-induced denaturation process, the protein in the gel system contains a large amount of moisture [20], which has a significant influence on the quality and yield of the final product [21]. In this study, the interaction between water and protein molecules was investigated using the LF-NMR technique, a nondestructive method for water mobility and distribution analysis through measuring the T_2_ relaxation time changes in the range from 38 to 90 °C. As shown in Figure 1a, three proton populations were found in the contour plots of T_2_ relaxation times for SP solution, which were defined as T_21_, T_22_, and T_23_, respectively. The first component (T_21_) was assigned to the highly integrated water located in protein macromolecules with a relaxation time of approximately 1.15 ms, at the initial temperature 38 °C. The second signal, T_22,_ with a relaxation time of 2.20 to 38.00 ms, was regarded as the inter-myofibrillar water, which was highly trapped in the organized protein structure [20]. The protons of the T_21_ and T_22_ components tightly attached to the protein molecules could not easily migrate during the gelation process. The peak areas of T_21_ and T_22_ (A_21_ and A_22_) fluctuated in the range of 7.92 to 141.80 and 2.97 to 36.25, respectively. The third component of T_23_ with a relaxation time of around 100 ms was ascribed to the extra-myofibrillar water [22]. The water mobility reflects the relationship between protons (mainly from water) and the biomacromolecules in the protein system. The protons with a shorter relaxation time had lower water mobility, which showed stronger binding ability to protein in contrast to those with a longer relaxation time. The higher water mobility means that the water molecules are not tightly attached to the protein backbone, which have longer spin-spin relaxation time (T_2_). Using this property, the gelation of the SP and MP from clam are dynamically assessed during the heating process. It is noteworthy that the temperature exhibited a significant impact on the relaxation profile of MP during the increase of temperature, which significantly influenced the formation of protein gelation [23]. No thermal gelation was formed for the SP:MP ratio of 10:0, 8:2, and 6:4 (Figure 1a–c), whereas significant peak splitting of T_23_ peak was observed during the gel formation (Figure 1e–g). This phenomenon can be used for real-time monitoring of water mobility and gelation change in the protein system. The gelation behavior occurred at 70, 62, and 52 °C after increasing the MP proportion of SP:MP to 4:6, 2:8, and 0:10, respectively. The temperature corresponding to peak splitting demonstrated that the gel formation of the dual-protein system and the gelation temperature decreased when the proportion of the clam MP increased. Moreover, the proton population of T_24_ appeared with an increase of temperature, which was assigned to the increased free water within the extra-myofibrillar space. It is known that the MP within meat batter has an NMR signal in the range of 100 to 1000 ms [24], and heating results in a decrease of T_23_ and an increase of T_24_ in pork MP [25]. Herein, the water distribution was directly visible as the proportion and temperature changed using contour plots. This is very applicable for monitoring water dynamic change during the gelation of the dual-protein system.

**Figure 2:**
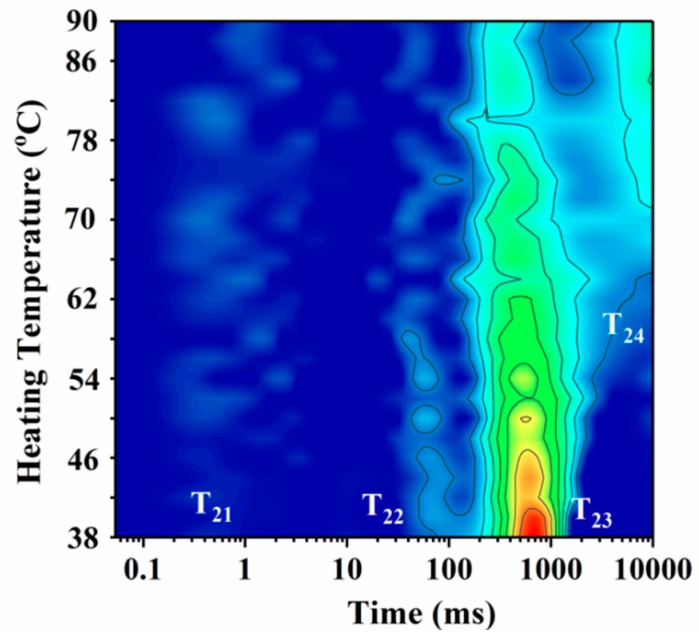
Moisture distribution change of MP-only system by adding 20% (*v*/*v*) distilled water at various temperatures.

To assess the volume effect on the gel formation temperature, taking the reaction condition of Figure 1e as an example, 20% SP was replaced by adding the same amount of distilled water (Figure 2). A right shift was observed for the contour plots of T_2_ relaxation times of MP solution upon addition of 20% (*v*/*v*) water in 80% MP during an increase of temperature from 38 to 90 °C. This indicated increased water mobility due to the introduction of free water [26]. The peak splitting occurred around 62 °C, similar to the result shown in Figure 1e (SP:MP = 2:8), suggesting that the SP concentration had little influence on MP gelation temperature. However, the addition of 20% SP significantly decreased water mobility of the dual-protein system because of strong retention of SP with moisture, as well as the interaction with surf clam MP (Figure 1e).

The relaxation time and proportions of each population in the dual-protein system before and after heating (90 °C) with different SP/MP components are shown in Table 1. There is no significant difference of T_2_ and A_2_ among the unheated samples. The relaxation time of T_23_ decreased with an increase of MP proportion, and the corresponding peak area proportion P_23_ was not changed without the appearance of the T_24_ component. This indicated that the mobility of the dual-protein system decreased, and the molecules assembly occurred. In contrast, the heated samples of the SP/MP system showed a dramatic change of T_23_ ranging from 77.27 to 396.40 ms, which was significantly shortened in the sample with SP:MP proportion at 2:8 and 0:10. Meanwhile, the A_23_ declined dramatically from over 80% to 31.88% or 42.74%, and the peak of T_24_ appeared with a relative value of 55.16% and 34.22%, respectively, for the 80% and 100% MP. This result demonstrated that the gelation occurred in the heated samples when the proportion of MP was more than 80%.

**Figure 3:**
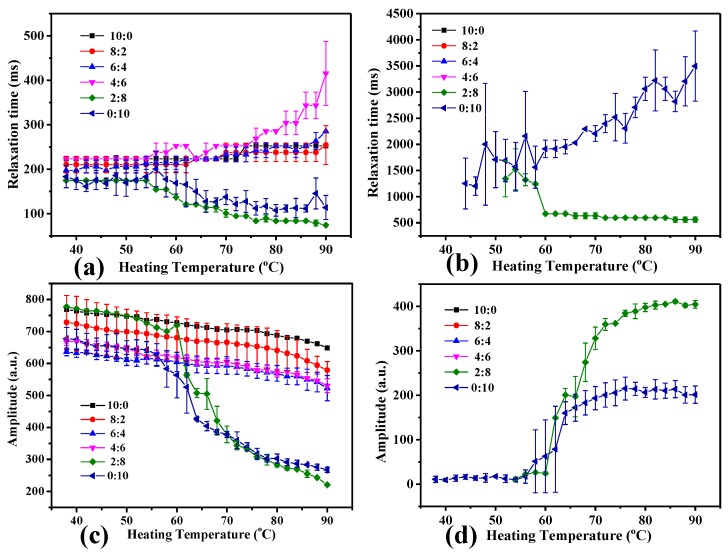
(**a**) T_23_; (**b**) T_24_; (**c**) A_23_; and (**d**) A_24_ changes of the SP and MP system during heating from 38 to 90 °C.

Figure 3 shows the change of relaxation time (T_23_ and T_24_) and corresponding peak area of T_23_ (A_23_) and T_24_ (A_24_) of the dual-protein system at different proportions of SP and MP. The T_23_ and A_23_ remained unchanged between 38 and 54 °C and exhibited different trends from 56 to 90 °C for the dual-protein gels with various SP and MP compositions. In the gel with SP:MP proportion a1t 0:0, 8:2, and 4:6, T_23_ increased mildly from 56 to 90 °C. When the MP proportion increased to 60% (SP:MP = 4:6), T_23_ drastically increased, revealing an increased water mobility. With a further increase of MP proportion to 80% or 100% (2:8 and 0:10), the relaxation time of T_23_ decreased gradually. Meanwhile, the T_24_ component appeared, indicating gelation occurrence (Figure 2b). T_24_ changed quickly with the decrease of T_23_, and the relaxation time changes indicated that the gelation formation started at 62 and 58 °C, respectively, for the dual-protein system with SP:MP proportion at 2:8 and 0:10. Compared with the single system of MP (0:10), the addition of a small amount of SP (SP:MP = 2:8) resulted in a lower T_24_ value. The A_23_ decreased with an increase of temperature from 38 to 90 °C, whereas A_24_ exhibited an increase trend during this process, suggesting the water mobilization was enhanced when the MP proportion increased. 

### 3.2. Principal Component Analysis (PCA)

**Figure 4:**
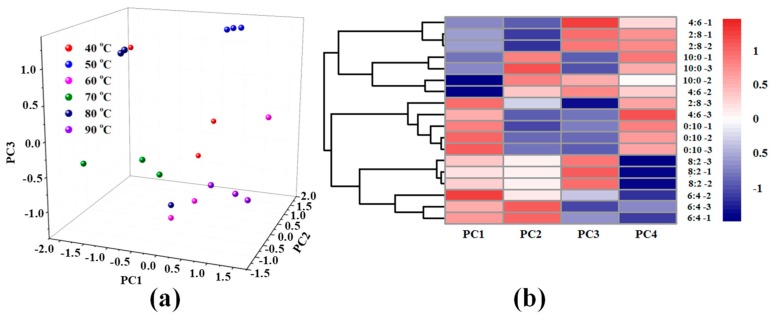
(**a**) Principal component analysis (PCA) and (**b**) heatmap of the SP and MP system with various MP/SP proportions at different heating temperatures.

Gel formation with various SP and MP proportions within 40 to 90 °C was analyzed with PCA by reducing the dimensions of the T_2_ relaxation information and distinguishing the samples of different heating temperatures (Figure 4). PCA is considered to be the fundamental method based on an eigenvector decomposition of the covariance matrix [27]. The complex data of T_2_ relaxation were reduced into several principal components for better presentation and analysis [28], in virtue of the water states and distribution for the little differences with temperature sets. The first three principal components (PC1 and PC2 versus PC3) explained 99.57% of the total variation in Figure 4a, and PC1 reached 66.23% total variation, and the training set off PC1 was not distinguished clearly according to the proton states in the protein system under different temperature conditions. PC2 explained 32.72% of the total variation which was mainly due to the variation between replicates. To better distinguish the mixed protein of different treated temperatures, a heatmap (Figure 4b) was used to treat the LF-NMR data, and higher scores are shown in red and lower scores in blue. In the heatmap, the gel with different SP and MP proportions was well grouped by PC1 and PC4, except the samples with SP and MP proportion at 10:0, which were randomly distributed in the heatmap. This result demonstrated that the PCA and heatmap have the potential to distinguish different dual-protein systems formed at various temperatures through analyzing the LF-NMR data.

### 3.3. Characterization of Rheology Property

**Figure 5:**
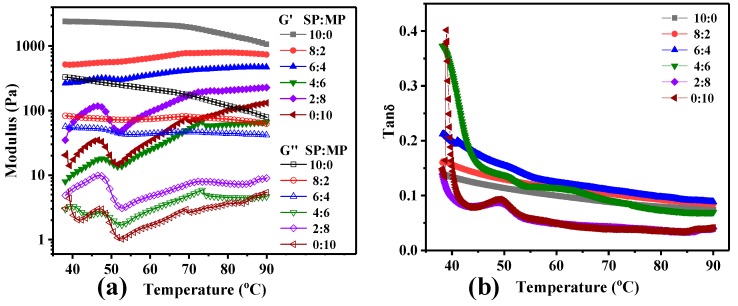
Rheology properties of (**a**) storage modulus G’ and loss modulus G’’; and (**b**) tan δ of the SP and MP system with various SP/MP proportions at different heating temperatures.

To evaluate the rheology properties of the dual-protein system with different SP and MP proportions during the heating process, the storage modulus G’ and loss modulus G’’ were recorded to evaluate elasticity and stickiness, respectively. The ratio of G’’ and G’ (tan δ) was used to estimate the viscoelasticity [29], which were related to the mobility of the mixed system. As shown in Figure 5a, the values of G’ and G’’ decreased when the proportion of SP:MP was 10:0, while G’ and G’’ almost remained unchanged when the proportion of SP:MP was 8:2 and 6:4. As the ratio of SP:MP was 4:6, 2:8, and 0:10, the G’ and G’’ showed an increased trend up to 47 °C, then, decreased until 52 °C, followed by a gradual increase to 90 °C. Meanwhile, the addition of MP decreased the elasticity and stickiness, while an increase of temperature caused an increase of viscosity. The protein systems formed a loose gel structure at a lower heating temperature less than 52 °C, and gradually increased to a strong gel. The ratio of G’’ and G’ also showed a broad peak around 49 °C and ending at 52 °C for the systems with SP:MP ratio 4:6, 2:8, and 0:10 (Figure 5b). This was also consistent with the generation of peak splitting around 52 °C observed for the T_2_ relaxation time profile in Figure 1, suggesting that the dual-protein samples containing MP gradually changed from sol to gel and more cross-links were formed during heating. The G’ have a synergistic result with surface hydrophobicity and network along with an increase of temperature. The intersection of G’ and G’’ is generally used to judge whether the gel is formed or not. However, it seems that the T_2_ spectra is much clearer for distinguishing gel formation. 

### 3.4. Cooking Loss, Water Holding Capability (WHC), and Gel Strength Analysis

**Figure 6:**
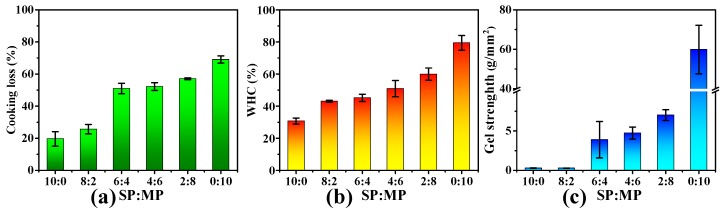
(**a**) Cooking loss; (**b**) water holding capacity (WHC); and (**c**) gel strength in the SP and MP system with various SP/MP proportions after heating at 90 °C for 1 h.

Physical indexes of the heated mixed system such as cooking loss, WHC, and gel strength are important factors for gel evaluation. The cooking loss, WHC, and gel strength of the dual-protein system are gradually improved with the addition of MP (Figure 6). The increase of cooking loss indicated that the moisture was excluded by the contracted network during formation of the gel. The WHC of the gel increased significantly, and the gel strength gradually increased to 239.44 g/mm^2^ in single MP system. This revealed that the SP and MP formed a strong network with an increase of the addition of clam protein. The heated sample of the dual-protein system showed that MP had a better gel property, and it played a major role in the gelation process. The gel of Mesona Blumes gum and rice starch also exhibited good WHC in sausage, and the use of dual-protein gel could be a potential replacement for fat in making low-fat and high-protein food items [30].

**Figure 7:**
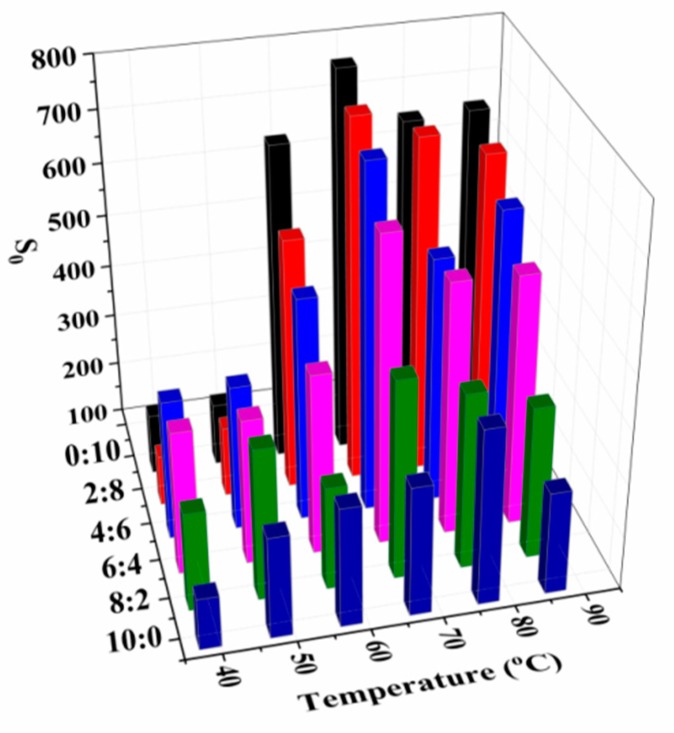
Hydrophobicity (S_0_) of the SP and MP system with various SP/MP proportions at different temperatures.

Figure 7 shows the hydrophobicity of the SP and MP system during the gelling process, which further explained the formation of protein network. The hydrophobicity started to increase for the samples with SP/MP proportions of 4:6, 2:8, and 0:10, when the temperature was over 50 °C. The hydrophobicity increased to a maximum value at 70 °C and, then, decreased until 90 °C. The protein molecules stretched after being heated at lower temperatures, resulting in the loss of tertiary and quaternary structure and, then, the buried hydrophobic groups were exposed in the external regions of protein in the aqueous environment. Thus, the hydrophobicity of protein surface increased and caused fluorescent probe ANS binding with the hydrophobicity parts of the protein molecules. However, excessively high temperatures led to contraction of the formed network and squeezed out part of the water with some soluble protein and reduced the hydrophobicity of the system. This result demonstrated that the reduced water mobility monitored by LF-NMR for dual-protein systems with SP/MP proportions of 4:6, 2:8, and 0:10 after heating was well explained by the hydrophobicity experiment. 

### 3.5. Microstructure of Mixed Protein

**Figure 8:**
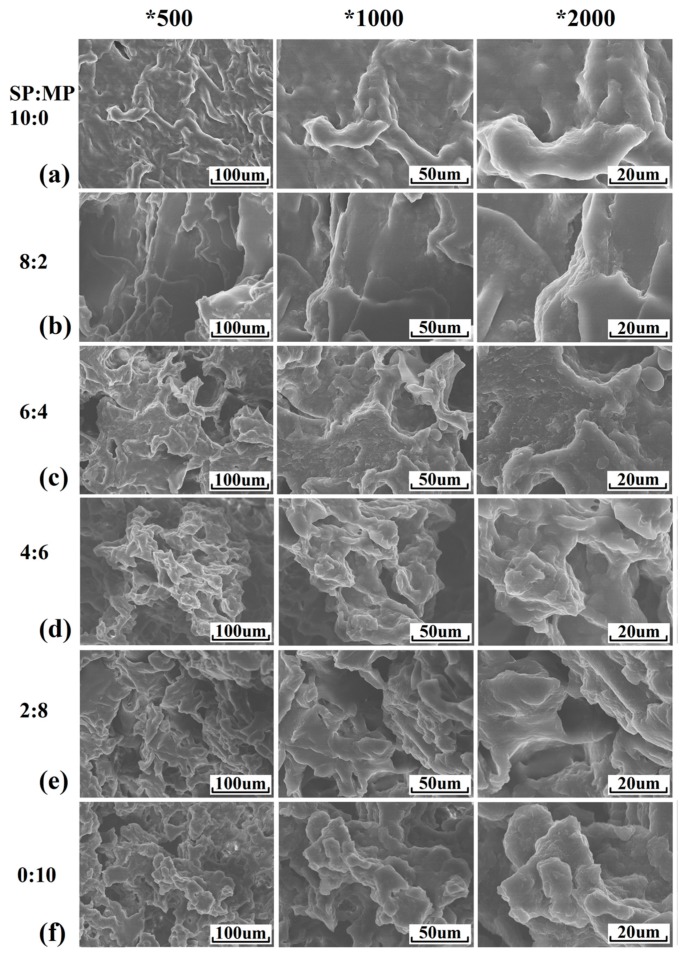
SEM graphs with different magnification times in the SP and MP system. SP/MP was set as 10:0 (**a**); 8:2 (**b**); 6:4 (**c**); 4:6 (**d**); 2:8 (**e**); and 0:10 (**f**).

SEM was applied to investigate the structure of the SP and MP protein system (Figure 8). The SP only (10:0) system appeared laminar structure, while the MP only (0:10) system was slightly porous. The number of micropores increased with the addition of MP for the dual-protein system of SP/MP. More micropores led to an improved water holding ability and enhanced the gel strength, which is observed in Figure 6. This phenomenon is probably due to the molecules rearrangement of the protein network after interaction of the SP and MP at higher temperatures, thus resulting in the formation of a cavity. Similar microstructure was also found in pork MP and SP mixed gels [31].

## 4. Conclusions

The current research indicated that the water mobility of a dual-protein SP and MP system can be characterized by LF-NMR during the gelation process. The moisture distribution showed a significant change in the dual-protein system with a higher MP content (SP:MP = 2:8) during heating. The dual-protein solution gradually translated into semisolid during heating and rheology results validated the formation of a network in the SP and MP system. The analysis results of cooking loss, gel strength, WHC, and hydrophobicity revealed that a higher percentage of MP contributed to the formation of the gel network, which significantly affected the moisture distribution. The SEM microstructure further provided evidence of the gelation changes for the different SP and MP proportion systems. Therefore, the LF-NMR can be a powerful tool for analyzing the water mobility changes of dual-protein systems. 

## Figures and Tables

**Table 1:** Low field nuclear magnetic resonance (LF-NMR) parameters of T_2_ relaxation times and peak area (P) of the dual-protein system with various SP/MP proportions before and after 90 °C heating for 1 h.

SP:MP	T_21_ (ms)	T_22_ (ms)	T_23_ (ms)	T_24_ (ms)	P_21_ (%)	P_22_ (%)	P_23_ (%)	P_24_ (%)
Before heating								
10:0	0.54 ± 0.31 ^a^	2.96 ± 0.54 ^b^	223.28 ± 0.00 ^a^	-	3.35 ± 0.82 ^a^	2.06 ± 1.17 ^a^	94.58 ± 0.50 ^a^	-
8:2	0.89 ± 0.26 ^a^	6.66 ± 2.41 ^b^	214.69 ± 14.88 ^a^	-	4.71 ± 1.35 ^a^	0.59 ± 0.29 ^a^	94.70 ± 1.59 ^a^	-
6:4	0.97 ± 0.55 ^a^	3.17 ± 1.09 ^ab^	206.10 ± 14.88 ^a^	-	4.53 ± 1.87 ^a^	0.97 ± 0.33 ^a^	94.49 ± 2.18 ^a^	-
4:6	0.61 ± 0.34 ^a^	4.30 ± 3.50 ^ab^	206.10 ± 14.88 ^a^	-	3.11 ± 2.69 ^a^	1.73 ± 1.50 ^a^	94.84 ± 1.34 ^a^	-
2:8	0.57 ± 0.28 ^a^	3.57 ± 1.05 ^a^	174.72 ± 0.00 ^a^	-	4.34 ± 0.62 ^a^	1.20 ± 0.30 ^a^	94.47 ± 0.92 ^a^	-
0:10	0.40 ± 0.05 ^a^	7.80 ± 2.44 ^a^	175.59 ± 21.49 ^a^	-	5.36 ± 0.45 ^a^	1.35 ± 0.21 ^a^	91.79 ± 0.36 ^a^	-
90 °C heating								
10:0	0.54 ± 0.14 ^ab^	5.66 ± 0.69 ^a^	252.42 ± 0.00 ^cd^	-	12.86 ± 2.23 ^ab^	2.29 ± 0.20 ^a^	84.85 ± 2.43 ^c^	-
8:2	0.49 ± 0.11 ^ab^	9.25 ± 1.13 ^a^	243.97 ± 35.84 ^c^	-	15.09 ± 1.91 ^ab^	2.61 ± 0.4a ^a^	82.30 ± 2.31 ^c^	-
6:4	0.89 ± 0.26 ^b^	15.85 ± 3.04 ^b^	285.35 ± 0.00 ^d^	-	15.49 ± 3.44 ^ab^	4.00 ± 0.41 ^a^	80.52 ± 3.34 ^c^	-
4:6	0.78 ± 0.31 ^ab^	9.05 ± 3.59 ^a^	396.40 ± 27.47 ^e^	-	14.22 ± 1.83 ^ab^	1.68 ± 0.21 ^a^	82.81 ± 1.46 ^c^	-
2:8	0.60 ± 0.04 ^ab^	5.26 ± 1.01 ^a^	77.27 ± 5.58 ^a^	526.86 ± 0.00	11.02 ± 0.83 ^a^	1.94 ± 0.44 ^a^	31.88 ± 0.60 ^a^	55.16 ± 1.36
0:10	0.38 ± 0.05 ^a^	8.01 ± 3.17 ^a^	126.19 ± 9.11 ^b^	2948.14 ± 360.89	18.71 ± 2.75 ^b^	4.32 ± 3.37 ^a^	42.74 ± 2.77 ^b^	34.22 ± 3.00

Values are presented as means ± standard deviation; different number superscripts indicate the significant difference (*p* < 0.05) between each treatment in a respective column.
